# Foreign

**Published:** 1841-04-24

**Authors:** 


### FOREIGN.

*Memoir of Sir Astley P. Cooper, Bart.*—Died, on the 12th Feb., in Conduit street, in his 73d year Sir Astley Paston Cooper, Bart., G. C. H., D. C. L., F. R. S., Sergeant Surgeon to the Queen. Sir Astley Cooper was the fourth son of Dr. Samuel Cooper, Rector of Great Yarmouth, Norfolk, and was born at Drooke, in the same county, August 23d, 1768. His mother, Susanna, was sprung from the ancient family of the Pastons, but was the daughter of James Bransby, Esq., of Shottisham, Norfolk, and was the authoress of a novel entitled *The Exemplary Mother.* In boyhood, Sir Astley is stated to have shown a bold and enterprising spirit, conjoined with a social disposition, and remarkable decision of character for so early an age: so truly

> "The childhood shows the man,
> As morning shows the day."

He received the common rudiments of his education from the village schoolmaster, Robert Larke; but his classical knowledge was derived from the instruction of his father, a good scholar, and the Rev. Joseph Harrison. At the age of fifteen, Sir Astley was placed with Mr. Turner, a surgeon and apothecary at Yarmouth, and was thus introduced to that profession of which he eventually became the brighest ornament. He, however, remained at Yarmouth but a few months, when he came to London, and was apprenticed to his uncle, Mr. William Cooper, one of the surgeons of Guy's Hospital; but with him he only remained three months, being then transferred, by his own desire, to Mr. Clive, the eminent surgeon of St. Thomas's Hospital. Sir Astley's labours in the dissecting-room were incessant—his attendance at the hospital, his examination of accidents, not less constant; and by this early zeal, united to his sound anatomical information, may be attributed the superiority he justly acquired in forming a diagnosis upon the nature of accidents, or disease. In after life, too, Sir Astley was wont to refer to this activity as an example of the habits of industry which he sought to impress upon thousands of pupils.

In 1787, Sir Astley visited Edinburgh for a short time; and though then scarcely of age, he honourably distinguished himself at the Royal Medical Society. Upon his return to London his master, Mr. Clive, appointed him demonstrator of anatomy, and soon after gave to him a part of the anatomical lectures. Sir Astley, also, with the consent of Mr. Clive and the other surgeons of St. Thomas's and Guy's Hospitals, gave a course of lectures on the principles and practice of surgery, which had previously only formed part of the anatomical course; and these lectures were really the foundation of his fame and fortune. It is true that this originality did not at first promise well, for his class consisted but of fifty students, though they eventually increased to four hundred, which was by far the largest number ever known in London. Of these lectures he gave a share to Mr. Travers, Mr. Henry Clive, and Mr. Joseph H. Green, consecutively. A little practice soon rendered him a popular teacher. He made no attempts at oratory; but was plain and practical in his details, and very successful in his illustrations; and he carefully avoided the introduction of controversial subjects connected with physiological science. At the expiration of his apprenticeship, in 1791, Sir Astley commenced as a lecturer. At the close of this year he married the daughter of Thomas Cock, Esq., of Tottenham, a distant relation of Mr. Clive; and, to show how solicitous he was never to neglect the performance of any public professional duty, it may be told, that on the evening of his wedding-day he delivered his customary lecture, without any knowledge of his marriage having been communicated to his class.

In the year 1792, Sir Astley visited Paris, and made himself master of the theory and practice of French Surgery. He used to relate that on August 10, while attending an operation, the fire of cannon announced the attack of the revolutionary mob upon the Tuileries, when he immediately ran upon the Pont Neuf, whence he could see the Swiss guards firing from the palace windows upon the people below. To reach home, he had to pass through the streets near the Palais Royal, amidst the roar of cannon, the firing of muskets, women bewailing the loss of their relatives, and crowds of men carrying upon pikes the heads or limbs of their victims. He several times heard Brissot, Marat, and Robespierre address the legislative assembly, and was once at the Jacobin Club. In later days Sir Astley frequently visited Paris, and became acquainted with Dupuytren, the celebrated surgeon, by whom he was introduced to Louis Phillippe, the Duke of Orleans, who subsequently gave Sir Astley the Cross of the Legion of Honour. He was soon after made an honorary member of the National Institute.

Upon his return to London, in 1792, Sir Astley commenced practice in Jeffrey-square, St. Mary Axe, where he lived six years; thence he removed to New Broad street, where he remained until 1815; when, from the great extension of his practice among the circles of the aristocracy, he removed to Spring gardens, and subsequently to Conduit street. Sir Astley's practice was now at its zenith, and his annual receipt of fees far exceeded that of any one member of the profession. In one year, the last of his residence in Broad street, he received no less than 21,000*l*. For many years after, his annual receipt was 15,000*l*., and upwards; his patients comprised all classes of society, and his attention was equally bestowed on the wealthy and the indigent.

In 1821, Sir Astley was created a baronet. He continued to lecture at St. Thomas's Hospital until 1826, the period of his lectureship having thus extended to thirty-five years. He was elected President of the Royal College of Surgeons for the years 1836 and 1837. He also delivered a course of Lectures on Comparative Anatomy at the College, of which he was likewise a member of the council and board of examiners.

In 1827, Sir Astley was appointed Sergeant Surgeon, which office he held at the time of his death. He was also surgeon to George IV; he attended William IV when he was first Lord of the Admiralty, and the Earl of Munster when he had a severe compound fracture of his leg. At the request of the Duke of Wellington, Sir Astley was made Grand Cross of the Guelphic Order. The University of Oxford conferred upon him the degree of Doctor of Civil Law; and numerous foreign academies eagerly enrolled his name among their members. He was likewise a fellow of the Royal Society, and of many scientific institutions in this country.

This accumulation of honours and fortune did not, however, induce Sir A. Cooper to relax in his practice, or in anatomical physiological inquiries—the results of which, to the astonishment of all acquainted with his daily labours, he had published upon a magnificent scale, but at a low price. The production of these works must have been very costly, for they abound with coloured engravings. But Sir Astley had a higher motive than pecuniary advantage in the publication of these works, namely: the mitigation of the sufferings of his fellow creatures. In his admirable, manly style, he said: "After having been for forty years placed in a situation of ample opportunity—after having been fostered by the profession and the public infinitely beyond my deserts, I feel that I only perform my duty in giving to my medical brethren, without any sordid views, the benefit of my experience." An excellent *precis* of these invaluable labours, extending to twenty-nine volumes, or important contributions, will be found in a memoir of Sir Astley Cooper, in Pettigrew's *Medical Portrait Gallery*, Parts V and VI; whence the preceding details have been chiefly derived. Sir Astley's first compositions appear to have been two contributions to a volume of Medical Researches, published in 1798; his last published work we believe to be the second part of *Illustrations of the Diseases of the Breast*, the first portion of which appeared in a handsome quarto volume, in 1829. Sir Astley Cooper, when most engaged in lectures and the practice of his profession, uniformly made it a rule to enter in his case book all the interesting operations and cases which he witnessed; and these books have been regularly preserved from 1800, with occasionally some prior to that period, as far back as 1784.

For some few years past Sir Astley Cooper had been anxious to relinquish a portion of his practice; but so eminent a practitioner could but ill be spared either by the public or the profession, and by all ranks of society his loss will be deeply lamented. Such retirement as Sir Astley could enjoy, he usually passed at a beautiful seat in the valley of the Gade, near

Hemel-Hempstead, in Hertfordshire, at a short distance from the London and Birmingham railway. Sir Astley had possessed this property for many years, and was otherwise a considerable landowner in the neighbourhood: here, as in public life, he was unsparing of his time or talents, for he was accessible by his neediest neighbour, and we believe that, for a long period, he was equally liberal to the poor of the metropolis. Sir Astley was much attached to farming pursuits, and at the period we have just referred to, he indicated personally none of the cares of professional study, but was, on the contrary, the *beau ideal* of a country gentleman.

Sir Astley Cooper's last illness was but of short duration. His fine athletic figure, and his known temperate and careful habits, probably, led his friends to regard him but as in green old age. It appears that on January 24, of the present year, from having taken a walk without any more than the ordinary precaution against the inclemency of the weather, Sir Astley was seized with difficulty of breathing, and pains about the chest. Dr. Bright and Mr. Bransby Cooper were in immediate attendance; but, notwithstanding the remedies administered, the disease continued to make further progress. On the Sunday following, Dr. Chambers was associated with the other medical attendants, and the report of the following day was much more favourable. This change was, however, but of short duration, and Sir Astley grew worse, until on Friday, the 12th February, when, somewhat suddenly, towards the termination, he sank.

By his marriage with Miss Cock, Sir Astley Cooper had only one daughter, who died at the early age of two years. Lady Cooper died in June, 1827; and in July, 1828, Sir Astley again entered the married state with Catherine, daughter of John Jones, Esq., of Derry Ormond, Cardiganshire; by whom he had no issue. The baronetcy, however, having been conferred with remainder, in default of male issue, to Astley Paston, the fourth son of his second brother, the Rev. S. L. Cooper, the title descends to that gentleman. Sir Astley is stated to have died worth upwards of half a million sterling. His museum is, perhaps, the most valuable private collection in Europe, and contains almost all the specimens which have furnished the magnificent illustrations of his published works. Of Sir Astley Cooper's professional life are related many interesting anecdotes, which alike bespeak his admirable nature and consummate skill; and, in all the relations of life, he is known to have presented a rare combination of the excellences of head and heart.

---

*Kiesteine.*—M. Nauche has given this name to a particular product of a gelatino-albuminous nature, which is found in the urine of women, at the end of the first month of pregnancy. This principle is separated by repose alone, from the other matters of the urine. It is sufficient to place this fluid in a glass; at the end of several hours the kiestine shows itself upon the surface, under the form of points of oblong filaments, which unite in a layer a line in thickness. A portion of this layer is precipitated to the bottom of the vessel, and there forms a white deposit, of a milky appearance. Another remains upon the surface of the urine, contracts adhesions with the sides of the vessel, and is converted into a solid membraniform substance.

The kiesteine furnishes a positive means of detecting pregnancy, even at its commencement, when the woman is in health. In diseases with purulent secretion, in the different dropsies, diabetes, in children with worms, the urine is often covered with an albuminous, fatty, or saline layer, which has some resemblance to that observed in pregnancy, but with a little practice, they are distinguished at the first glance. The existence of these diseases ought, moreover, to put us upon our guard against all mistakes of this kind.

M. Nauche assures us, that he has frequently employed this method of determining pregnancy, in the case where it was far from being expected. He thinks that kiesteine is also found in animals under the same circumstances.

Emanuel Russeau has undertaken, at the Garden of Plants, experiments upon the same subject. He has found the same substance as M. Nauche in the urine of apes during gestation. He has in vain sought for it in those who were not fecund—or in the urine of males.

*Journ. de Chemie Med.*.

---

NEW REMEDY.

*Johnson's Syrup.*—The patent granted to the inventor for five years having expired, this syrup has become public property. It consists of an ounce of syrup of liquorice, forty-six grains of the fresh juice of asparagus heads, and the same quantity of the syrup of mallow.

---

*Poisoning in rabbits by large doses of Sulphate of Quinine.* By M. Desiderio.—The author, in a paper written in Italian, makes known the results of nine experiments in which he gave rabbits the sulphate of quinine, either alone or united with other substances. In the two first experiments, forty grains given to adult animals produced death in less than five hours. A young rabbit was killed in six hours by fifteen grains. In the other experiments the author associated with the action of the sulphate of quinine, that of alcohol weakened with water of canella, that of distilled water of the *Laurus cerasus*, and, finally, that of opium.

*Journ. de Chem. Med.*

